# Cascaded Photon Upcycling in an Upconversion-Plasmonic Fabry-Pérot Cavity for Broadband Solar Hydrogen Production from PLA Waste

**DOI:** 10.3390/ma19142994

**Published:** 2026-07-11

**Authors:** Longhui Han, Jingyuan Zheng, Kaiqi Li, Yang Li, Yaru Ni, Chunhua Lu

**Affiliations:** 1 State Key Laboratory of Materials-Oriented Chemical Engineering, College of Materials Science and Engineering, Nanjing Tech University, Nanjing 210009, China; hlh1691553469@163.com (L.H.); 202362031031@njtech.edu.cn (J.Z.); kaiqili@njtech.edu.cn (K.L.);; 2 Jiangsu Collaborative Innovation Center for Advanced Inorganic Function Composites, Nanjing Tech University, Nanjing 210009, China; 3 Jiangsu National Synergetic Innovation Center for Advanced Materials (SICAM), Nanjing Tech University, Nanjing 210009, China

**Keywords:** plasmonic nanostructures, Fabry-Pérot cavity, photocatalytic hydrogen evolution, broadband light utilization, upconversion

## Abstract

Solar-driven hydrogen evolution is limited by the poor ability of conventional photocatalysts to utilize the visible–near-infrared (Vis–NIR) region, which accounts for ~95% of the solar spectrum. Here, we design an upconversion-plasmonic Fabry–Pérot cavity (Al/NaYF_4_:Yb^3+^,Tm^3+^/Au/TiO_2_) to achieve cascaded photon upcycling for efficient solar hydrogen production. In this architecture, the NaYF_4_:Yb^3+^,Tm^3+^ layer serves as the dielectric medium of the cavity, enabling multiple light reflections and enhanced NIR-to-UV/Vis upconversion. The upconverted photons, together with the incident light, are further concentrated by the adjacent Au layer via surface plasmon resonance, promoting hot-electron generation and injection into TiO_2_. As a result, the optimized structure achieves a broadband absorption efficiency of 60.57% and a hydrogen evolution rate of 19.86 mmol·g^−1^·h^−1^ from polylactic acid wastewater, 124 times higher than that of pristine TiO_2_. This work provides a scalable strategy for broadband solar harvesting and plastic-waste-to-hydrogen conversion.

## 1. Introduction

Global plastic pollution has reached a critical level, with annual waste generation exceeding 400 million metric tons [1]. Polylactic acid (PLA), despite being marketed as “biodegradable”, degrades slowly in natural environments and now represents a growing fraction of this waste stream, leading to persistent accumulation [2]. Conventional recycling methods, such as thermal pyrolysis, are energy-intensive and often generate secondary pollutants, highlighting the urgent need for more sustainable solutions [3,4].

In this context, solar-driven photocatalytic hydrogen production from PLA wastewater has emerged as a promising strategy to simultaneously address plastic pollution and clean energy demands [5,6,7,8]. However, the efficiency of this process is fundamentally limited by the severe spectral mismatch between sunlight and conventional wide-bandgap photocatalysts like TiO_2_ [9,10]. These materials can only utilize ultraviolet (UV) photons, which constitute a mere ~5% of the solar spectrum, while the vast majority of energy in the visible (Vis, ~43%) and near-infrared (NIR, ~52%) regions remains unharnessed [11]. Harnessing this abundant low-energy light thus represents a central challenge.

Plasmonic metals, such as Au, provide a potential route for extending photocatalytic activity into the Vis spectral ranges [12]. This is achieved through surface plasmon resonance (SPR), where free electrons in the metal collectively oscillate under light excitation, generating highly energetic “hot” electrons [13,14]. In contrast to the bandgap limitation of semiconductors, this plasmonic mechanism enables the direct utilization of low-energy photons. This process involves the resonant excitation of electrons near the metal Fermi level, producing energetic hot carriers above the Fermi level that can directly drive surface reactions without relying on interband excitation across a semiconductor bandgap [15]. However, these hot electrons exhibit an extremely short lifetime (ps-fs scale), necessitating efficient injection across the metal/semiconductor interface into the semiconductor, where their lifetime can extend to nanoseconds or even milliseconds, to participate in photocatalytic reactions at active sites [16,17]. A major limitation arises in the NIR region, where the efficiency of hot-electron injection declines precipitously due to insufficient photon energy [18]. Upconversion materials provide a complementary strategy by converting multiple low-energy NIR photons into a single high-energy UV/Vis photon [19,20]. While integrating upconversion materials with plasmonic systems is conceptually attractive, conventional fabrication strategies, such as powder-type or core–shell NaYF_4_-based photocatalysts coupled with TiO_2_ or plasmonic metals to extend the NIR photoresponse, fail to achieve the nanoscale spatial precision required for efficient, sequential energy transfer, leading to significant optical losses and suboptimal performance [21,22,23].

**Figure 1:**
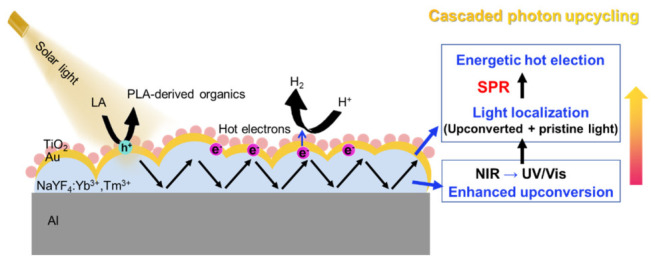
Schematic of the cascaded photon-upcycling system based on an upconversion−plasmonic F−P microcavity (Al/NaYF_4_:Yb^3+^,Tm^3+^/Au/TiO_2_) for enhanced broadband solar−driven hydrogen production.

Here, this work designs a cascaded photon-upcycling system based on an upconversion-plasmonic Fabry-Pérot (F-P) cavity (Al/NaYF_4_:Yb^3+^,Tm^3+^/Au/TiO_2_, Figure 1). The cavity is formed by the Al and Au reflective layers with the central upconversion dielectric medium (a layer of multisized NaYF_4_:Yb^3+^,Tm^3+^ nanoparticles) sandwiched between them, trapping incident light, forcing multiple passes through the upconversion layer to enhance NIR-to-UV/Vis conversion [24,25,26]. The adjacent Au plasmonic layer concentrates both the upconverted photons and the pristine incident light via SPR, where this enhanced localized light promotes the generation of energetic hot electrons for subsequent injection into the nearby TiO_2_ photocatalyst to drive the hydrogen evolution reaction. This sequential photon management achieves a broadband absorption efficiency of 60.57% (a 31.08% enhancement over a bare Au nanofilm) and a remarkable hydrogen evolution rate of 19.86 mmol·g^−1^·h^−1^ from PLA wastewater, which is 124 times higher than that of pristine TiO_2_. This work thereby establishes an effective photon-upcycling pathway for broadband solar energy harvesting and sustainable hydrogen production from plastic waste.

## 2. Experimental Section

### 2.1. Materials and Reagents

Sodium fluoride (NaF, 99.99%), ytterbium fluoride (YbF_3_, 99.99%), yttrium fluoride (YF_3_, 99.99%), and thulium fluoride (TmF_3_, 99.99%) were purchased from MERYER (Shanghai, China) and used as precursor materials for the synthesis of upconversion phosphors. Aluminum (Al, 99.99%) and titanium dioxide (TiO_2_) sputtering targets were obtained from Infision Optoelectronics (Fuzhou, China), while the gold (Au, 99.999%) target was supplied by Hangdan Optoelectronics (Hangzhou, China). Polylactic acid (PLA) was purchased from Beijing InnoChem Science & Technology Co., Ltd. (Beijing, China). Anhydrous ethanol (C_2_H_5_OH, 99.7%) was purchased from Nanjing Wanqing Chemical Glass Instrument Co., Ltd. (Nanjing, China).

### 2.2. Preparation of Multisized NaYF_4_:Yb^3+^,Tm^3+^ Nanoparticles

To prepare the multisized NaYF_4_:Yb^3+^,Tm^3+^ nanoparticles, bulk upconversion powders were synthesized and subsequently milled (Figure S1). First, the NaYF_4_:Yb^3+^,Tm^3+^ powders were synthesized by a conventional high-temperature solid-state reaction. Stoichiometric mixtures of NaF, YbF_3_, YF_3_, and TmF_3_ (molar ratio = 1:0.835:0.15:0.015) were thoroughly ground for 1.5 h and then calcined at 900 °C for 6 h under an argon atmosphere. Subsequently, the as-synthesized NaYF_4_:Yb^3+^,Tm^3+^ powder was subjected to high-energy ball milling. The milling was performed in a planetary ball mill at a rotational speed of 300 rpm with a ball-to-powder mass ratio of 5:1, using 60 g of anhydrous ethanol as the dispersion medium, and the milling time was set to 3 h. After milling, the resulting slurry was centrifuged at 10,000 rpm for 5 min to achieve solid–liquid separation, and the solid product was collected. The collected solid was then dried in an oven at 80 °C to obtain the final dried powder sample.

### 2.3. Preparation of Upconversion-Plasmonic F-P Cavity

Preparation of Al/NaYF_4_:Yb^3+^,Tm^3+^/Au/TiO_2_: First, a 100 nm-thick Al layer was pre-deposited onto glass substrates to obtain glass/Al substrates. Subsequently, 50 mg of dried NaYF_4_:Yb^3+^,Tm^3+^ powder was dispersed in 20 mL of anhydrous ethanol and ultrasonicated for 15 min to form a homogeneous suspension. The glass/Al substrates were immersed in the above suspension and shaken on a mechanical oscillator for 30 min, allowing NaYF_4_:Yb^3+^,Tm^3+^ particles to uniformly attach to the substrate surface via electrostatic adsorption. After adsorption, the substrates were removed and dried in an oven to obtain a uniformly distributed NaYF_4_:Yb^3+^,Tm^3+^ functional layer (Al/NaYF_4_:Yb^3+^,Tm^3+^). Finally, the Al/NaYF_4_:Yb^3+^,Tm^3+^ substrates were transferred into a vacuum deposition system (TEMD500, Beijing Taikenuo Technology Co., Ltd., Beijing, China) for the sequential deposition of Au and TiO_2_ layers, yielding the Al/NaYF_4_:Yb^3+^,Tm^3+^/Au/TiO_2_ multilayer structure. Prior to deposition, the chamber was evacuated to a base pressure of 2 × 10^−3^ Pa. During deposition, the substrates were rotated at 10 rpm to ensure film uniformity. A 5 nm Au layer was first deposited at a rate of 0.1 Å s^−1^, followed by deposition of a 100 nm TiO_2_ layer at 0.3 Å s^−1^. All depositions were conducted at room temperature without intentional substrate heating. For consistency and comparability, all samples were prepared under identical vacuum conditions and deposition parameters.

Preparation of Al foil/NaYF_4_:Yb^3+^,Tm^3+^/Au/TiO_2_: First, aluminum foil (Al foil) was cut into the desired size as the substrate and sequentially ultrasonically cleaned in acetone, anhydrous ethanol, and deionized water (10 min each). The foil was then dried in an oven to obtain a clean Al foil substrate. Subsequently, 50 mg of dried NaYF_4_:Yb^3+^,Tm^3+^ powder was dispersed in 20 mL of anhydrous ethanol and ultrasonicated for 15 min to form a homogeneous suspension. The cleaned Al foil substrate was immersed in the above suspension and shaken on a mechanical shaker for 30 min, allowing NaYF_4_:Yb^3+^,Tm^3+^ particles to uniformly attach to the foil surface via electrostatic adsorption. After adsorption, the substrate was removed and dried in an oven to obtain a uniformly distributed NaYF_4_:Yb^3+^,Tm^3+^ functional layer (Al foil/NaYF_4_:Yb^3+^,Tm^3+^). Subsequently, the Al foil/NaYF_4_:Yb^3+^,Tm^3+^ substrate was transferred into a vacuum deposition system (TEMD500, Beijing Taikenuo Technology Co., Ltd.), where Au and TiO_2_ layers were sequentially deposited under the same vacuum conditions and deposition parameters as those used for the aforementioned Al/NaYF_4_:Yb^3+^,Tm^3+^/Au/TiO_2_ sample, yielding the Al foil/NaYF_4_:Yb^3+^,Tm^3+^/Au/TiO_2_ multilayer structure.

### 2.4. Characterization

Crystal structures were analyzed by X-ray diffraction (XRD, Rigaku Ultima IV, Rigaku Corporation, Tokyo, Japan). Surface morphologies were examined by scanning electron microscopy (SEM, ZEISS Sigma 360, Carl Zeiss AG, Oberkochen, Germany). Up-conversion photoluminescence (PL) spectra were recorded on a fluorescence spectrometer (FL3-221, HORIBA Jobin Yvon) under excitation with a 980 nm semiconductor infrared laser (500 mW). The basic optical properties of the films were measured using a UV-Vis spectrophotometer (Agilent Cary 5000, Edison, NJ, USA). The reflectance/transmittance spectra used to evaluate the spectral absorptance (A = 1-R-T) and to corroborate cavity-enabled light trapping are shown in Figure S2 (Supporting Information). In situ X-ray photoelectron spectroscopy (XPS, Thermo Fisher Scientific ESCALAB 250Xi, Waltham, MA, USA) was employed to probe interfacial carrier transfer: spectra were first collected after 10 min in the dark and then after 10 min under full-spectrum illumination (200–2500 nm). Binding-energy shifts of Ti 2*p*, O 1*s*, and Au 4*f* (together with changes in the valence-band maximum) were analyzed to infer carrier migration direction and recombination suppression at the interfaces.

### 2.5. Electrochemical Performances

Electrochemical measurements were performed in a standard three-electrode configuration, with a 0.5 cm × 1 cm sample film as the working electrode, a platinum foil as the counter electrode, and an Ag/AgCl (3 M KCl) electrode as the reference. The electrolyte was an aqueous 0.5 M Na_2_SO_4_ solution. Photocurrent-time (I-t) and electrochemical impedance spectroscopy (EIS) measurements were carried out on an electrochemical workstation (Autolab PGSTAT302N, Metrohm Autolab B.V., Utrecht, Switzerland) under illumination from a 300 W full-spectrum xenon lamp.

### 2.6. Photocatalytic H_2_ Evolution Tests

Photocatalytic hydrogen evolution experiments were conducted in a custom-built glass reactor connected to a closed gas-circulation system. A 300 W full-spectrum xenon lamp (200–2500 nm) was used as the light source. Prior to each reaction, the reactor was thoroughly purged with high-purity argon to remove residual air. The catalyst consisted of a 2.5 cm × 2.5 cm film sample immersed in an aqueous lactic acid solution with a concentration of 5 mg mL^−1^. PLA was pretreated by a hydrothermal method to prepare the substrate for subsequent degradation. Specifically, 1 g of PLA was added to a 100 mL Teflon-lined stainless-steel autoclave containing 50 mL of deionized water and heated at 200 °C for 1 h. The obtained PLA stock solution had a concentration of 20 mg mL^−1^ and was further diluted to 5 mg mL^−1^ for use as the reaction solution. During illumination, the evolved hydrogen gas was periodically sampled and quantified using a gas chromatograph (Agilent 7890B, Agilent Technologies, Santa Clara, CA, USA.) equipped with a thermal conductivity detector (TCD). Calibration was performed using standard gas mixtures to ensure accurate quantification. For the cycling stability tests, the optimized film catalyst was kept in the same PLA aqueous solution throughout the test. After each photocatalytic H_2_-evolution run, PLA was not reintroduced into the photoreactor. Instead, the reactor was re-purged with high-purity Ar to remove the generated gas and residual air before the next run. The H_2_-evolution test was then restarted under the same irradiation conditions, and this procedure was repeated for five consecutive cycles.

## 3. Results and Discussion

### 3.1. Synthesis and Optimization of Multisized Upconversion Particles

Bulk NaYF_4_:Yb^3+^,Tm^3+^ powders were synthesized via a high-temperature solid-state sintering method [27]. To identify the optimal luminescent center concentration, a series of samples with different rare-earth doping ratios (Yb^3+^:Tm^3+^ = 0.15:0.015, 0.20:0.020, and 0.25:0.025) were prepared [28]. A comparison of their upconversion emission spectra under 980 nm laser excitation (Figure S3, Supporting Information) revealed that the sample with Yb^3+^:Tm^3+^ = 0.15:0.015 exhibits the highest emission intensity, and therefore, this composition was selected for subsequent experiments.

**Figure 2:**
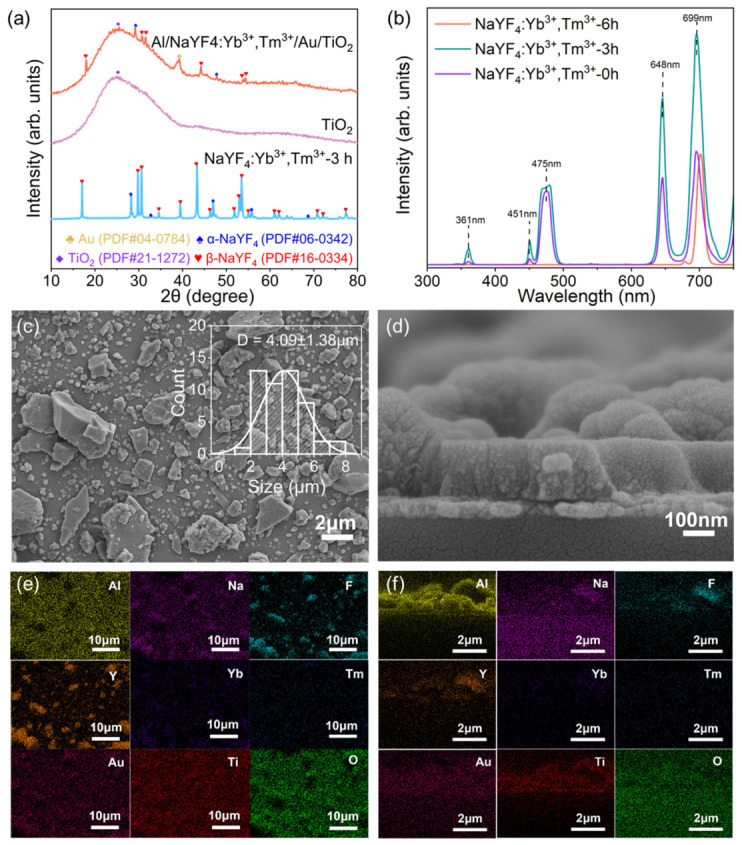
(**a**) X-ray diffraction (XRD) patterns of NaYF_4_:Yb^3+^,Tm^3+^–3h powder, TiO_2_ film, and the Al/NaYF_4_:Yb^3+^,Tm^3+^/Au/TiO_2_ multilayer structure. (**b**) Upconversion photoluminescence (PL) spectra of NaYF_4_:Yb^3+^,Tm^3+^ powders after 0 h, 3 h, and 6 h of ball milling (λ_ex_ = 980 nm). (**c**) Top–view SEM image. (**d**) Cross–sectional SEM image. (**e**) Top–view EDS mapping. (**f**) Cross–sectional EDS mapping.

To achieve broadband spectral response within the upconversion-plasmonic F-P cavity, the optimized NaYF_4_:Yb^3+^,Tm^3+^ (Yb^3+^:Tm^3+^ = 0.15:0.015) powder was subjected to ball milling to create a multisized particle distribution. This multisized architecture is crucial as it induces Mie scattering across a wide wavelength range, and the response wavelength is strongly dependent on the subwavelength particle sizes, enabling effective light trapping and interaction over a broad spectrum [29]. As shown in Figure 2a and Figure S4 (Supporting Information), no obvious peak shift was observed before and after milling, indicating that the powder remains predominantly in the hexagonal β-NaYF_4_ phase with a small fraction of the cubic α-NaYF_4_ phase. SEM-based particle-size statistics (Figure S5, Supporting Information) show that the particle size gradually decreases with increasing ball-milling time. Specifically, the characteristic particle size decreases from 6.54 ± 4.48 μm for the pristine powder (0 h) to 4.09 ± 1.38 μm after 3 h milling, and further to 2.79 ± 0.94 μm after 6 h milling. Notably, the 3 h milled sample still exhibits a broad size distribution (~0.75–7.75 μm), representing a typical multisized feature that can provide a particle-size basis for light scattering and optical coupling in subsequent broadband-responsive architectures. These results demonstrate that ball milling effectively tailors both the particle size and its distribution breadth while preserving the crystal framework of NaYF_4_:Yb^3+^,Tm^3+^.

In Figure 2b, the upconversion emission of NaYF_4_:Yb^3+^,Tm^3+^ powders was systematically examined under near-infrared excitation at 980 nm (λ_ex_ = 980 nm). Several characteristic Tm^3+^ upconversion emission bands are clearly resolved at approximately 361, 451, 475, 648, and 699 nm, which can be assigned to the ^1^D_2_ → ^3^H_6_, ^1^D_2_ → ^3^F_4_, ^1^G_4_ → ^3^H_6_, ^1^G_4_ → ^3^F_4_ and ^3^F_3_ → ^3^H_6_ transitions, respectively [30,31]. These emissions originate from the strong absorption of 980 nm photons by Yb^3+^ followed by stepwise energy transfer to Tm^3+^, which drives multiphoton upconversion processes (energy-transfer upconversion and/or excited-state absorption), thereby enabling NIR-to-Vis/UV photon conversion. Notably, the upconversion intensity reaches a maximum after 3 h of ball milling while the peak positions remain essentially unchanged. However, when the milling time is extended to 6 h, the blue emissions at 451 and 475 nm are markedly suppressed and the overall luminescence intensity decreases substantially. This behavior indicates that excessive milling introduces a high density of surface and lattice defects, which act as nonradiative recombination centers and lead to luminescence quenching [32]. Therefore, 3 h is identified as the optimal milling time, providing the best balance between particle-size reduction (and thus reduced light scattering) and the preservation of high upconversion efficiency. The resulting multisized powders were subsequently employed for the fabrication of the resonant cavity structures.

### 3.2. Fabrication and Structural Characterization of Upconversion-Plasmonic F-P Cavity

The Al/NaYF_4_:Yb^3+^,Tm^3+^/Au/TiO_2_ F-P cavity was fabricated through a stepwise process consisting of vacuum deposition of an Al reflective layer, electrostatic adsorption of NaYF_4_:Yb^3+^,Tm^3+^ particles (milled for 3 h), and sequential vacuum deposition of Au/TiO_2_. As illustrated in Figure 1, the resulting structure is a vertical multilayer stack: a thick Al layer serves as a highly reflective back mirror, the multisized NaYF_4_:Yb^3+^,Tm^3+^ particle layer acts as the photon-upconverting cavity spacer, and an ultrathin Au layer capped with TiO_2_ forms the top functional interface. This design efficiently traps incident light due to the high reflectivity of Al and the controlled optical path length. Simultaneously, the Au layer introduces plasmonic enhancement, working synergistically with TiO_2_ to achieve a broadband optical response and facilitate hot-electron injection for photocatalysis.

Compared with previously reported NaYF_4_-based upconversion photocatalysts, including powder-type and core–shell systems such as NaYF_4_:Yb^3+^,Er^3+^,Tm^3+^@TiO_2_–Ag composites and β-NaYF_4_:Yb^3+^,Tm^3+^/Er^3+^@SiO_2_@TiO_2_ photocatalysts, the present fabrication strategy is distinguished by the construction of a spatially ordered vertical multilayer cavity [33,34]. In those reported systems, the NaYF_4_-based upconversion component mainly served as a NIR-to-UV/Vis spectral converter and was coupled with TiO_2_ or noble-metal nanoparticles to enhance photocatalytic activity. However, they did not integrate an upconversion layer, plasmonic metal layer, and reflective back mirror into a Fabry–Pérot cavity. In contrast, the present Al/NaYF_4_:Yb^3+^,Tm^3+^/Au/TiO_2_ architecture arranges the Al reflector, multisized NaYF_4_:Yb^3+^,Tm^3+^ spacer, ultrathin Au plasmonic layer, and TiO_2_ photocatalytic layer in a defined vertical sequence, enabling cascaded light trapping, photon upconversion, plasmonic near-field enhancement, and interfacial electron injection. This spatially ordered fabrication process therefore provides a key structural basis for broadband photon management and enhanced photocatalytic H_2_ evolution.

The surface and cross-sectional morphologies of the Al/NaYF_4_:Yb^3+^,Tm^3+^/Au/TiO_2_ multilayer are shown in Figure 2c,d. The top-view scanning electron microscope (SEM) image indicates a continuous film with moderate surface roughness, which is beneficial for enhancing multiple light scattering and near-field optical interactions. The cross-sectional SEM image clearly resolves the multilayer architecture, including a dense Al reflective layer at the bottom, a continuous NaYF_4_:Yb^3+^,Tm^3+^ particle layer, an ultrathin intermediate Au layer, and a dense TiO_2_ capping layer. Elemental distributions via energy-dispersive X-ray spectroscopy (EDS) mapping are shown in Figure 2e,f. The results reveal clear spatial separation of the constituent elements along the thickness direction. Specifically, Na, Y, Yb, Tm, and F signals are concentrated in the NaYF_4_:Yb^3+^,Tm^3+^ layer, Au is confined to a narrow region at the NaYF_4_:Yb^3+^,Tm^3+^/TiO_2_ interface, and Ti and O are mainly distributed in the outer TiO_2_ layer. SEM observations further confirm that the deposited TiO_2_ consists of uniformly distributed particles that pack together to form a continuous film, and the corresponding EDS show a homogeneous distribution of Ti and O across the surface (Figure S6). This layered sequence confirms the successful fabrication of the designed upconversion-plasmonic F-P cavity.

The as-fabricated Al/NaYF_4_:Yb^3+^,Tm^3+^/Au/TiO_2_ vertical multilayer stack employs Al as a back reflector, a NaYF_4_:Yb^3+^,Tm^3+^ particle layer as the functional spacer, and subsequently deposited ultrathin Au and TiO_2_ capping layers, enabling cooperative optical coupling between the cavity resonance and plasmonic excitation. As shown in Figure 2a, the corresponding XRD patterns reveal a broadened diffraction halo centered at ~25° for the TiO_2_ top layer deposited at room temperature, indicating a predominantly amorphous/poorly crystalline nature. In contrast, the Au-containing sample exhibits a distinct diffraction peak at ~38.2°, which can be indexed to the Au (111) plane, confirming the crystalline character of the ultrathin Au layer. More importantly, the characteristic diffraction peaks of NaYF_4_:Yb^3+^,Tm^3+^ remain clearly observable in the multilayer structure, demonstrating that the NaYF_4_:Yb^3+^,Tm^3+^ particles preserve good phase integrity and structural stability during the subsequent vacuum deposition of Au/TiO_2_.

The crystallite sizes of the individual crystalline components were further estimated from the XRD data using the Scherrer equation, D = Kλ/(βcosθ) [35], where (D) is the crystallite size, (K) is the shape factor, (λ) is the X-ray wavelength, (β) is the full width at half maximum of the selected diffraction peak, and (θ) is the Bragg angle. As summarized in Table 1, the crystallite sizes of TiO_2_ and Au were calculated to be 6.6 and 7.3 nm, respectively, based on the TiO_2_ (101) and Au (111) diffraction peaks. The crystallite sizes of α-NaYF_4_:Yb^3+^,Tm^3+^ and β-NaYF_4_:Yb^3+^,Tm^3+^ were estimated to be 55.6 and 53.2 nm, respectively, indicating their relatively well-developed crystalline domains. The nanoscale TiO_2_ and Au crystallites are favorable for constructing abundant interfacial contact between the plasmonic metal and semiconductor, thereby facilitating hot-electron injection and interfacial charge transfer. Meanwhile, the highly crystalline NaYF_4_:Yb^3+^,Tm^3+^ layer can serve as an efficient upconversion medium and dielectric spacer in the Fabry–Pérot cavity. These structural features provide a solid basis for the cascaded photon-upcycling process in the Al foil/NaYF_4_:Yb^3+^,Tm^3+^/Au/TiO_2_ multilayer photocatalyst.

Overall, these results demonstrate the successful fabrication of the designed Al/NaYF_4_:Yb^3+^,Tm^3+^/Au/TiO_2_ multilayer structure.

### 3.3. Optical Behaviors of Upconversion-Plasmonic F-P Cavity

**Figure 3:**
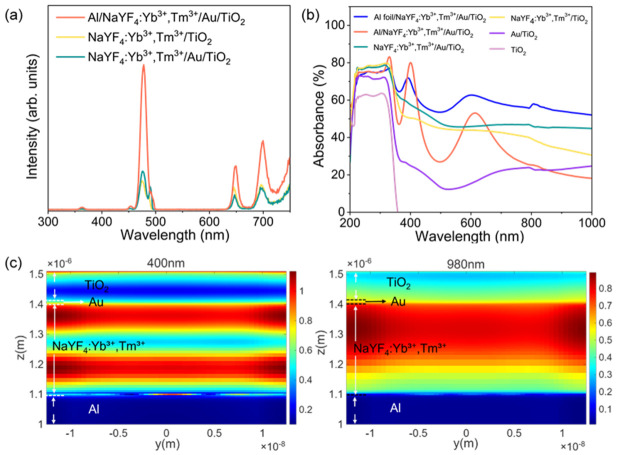
(**a**) Up-conversion emission spectra (λ_ex_ = 980 nm) of NaYF_4_:Yb^3+^,Tm^3+^/TiO_2_, NaYF_4_:Yb^3+^,Tm^3+^/Au/TiO_2_, and Al/NaYF_4_:Yb^3+^,Tm^3+^/Au/TiO_2_. (**b**) UV−Vis absorption spectra of TiO_2_, NaYF_4_:Yb^3+^,Tm^3+^/TiO_2_, Au/TiO_2_, NaYF_4_:Yb^3+^,Tm^3+^/Au/TiO_2_, Al/NaYF_4_:Yb^3+^,Tm^3+^/Au/TiO_2_, and Al foil/NaYF_4_:Yb^3+^,Tm^3+^/Au/TiO_2_. (**c**) FDTD−simulated normalized electric-field intensity maps (|E|^2^) of the Al/NaYF_4_:Yb^3+^,Tm^3+^/Au/TiO_2_ multilayer at incident wavelengths of 400 and 980 nm.

The influence of the upconversion–plasmonic F–P cavity on the optical behavior was investigated by means of upconversion photoluminescence spectroscopy, UV–Vis absorption spectroscopy, and finite-difference time-domain (FDTD) electric-field simulations. All three samples (NaYF_4_:Yb^3+^,Tm^3+^/TiO_2_, NaYF_4_:Yb^3+^,Tm^3+^/Au/TiO_2_, and Al/NaYF_4_:Yb^3+^,Tm^3+^/Au/TiO_2_) exhibit the characteristic upconversion emission features of Tm^3+^ under 980 nm excitation, with nearly identical spectral profiles and peak positions. As shown in Figure 3a, the emissions are mainly located in the blue and near-red/near-infrared regions. The blue bands at approximately 451 and 475 nm can be assigned to the Tm^3+^ transitions of ^1^D_2_ → ^3^F_4_ and ^1^G_4_ → ^3^H_6_, respectively. In the red/near-infrared region, emission bands at about 648 and 699 nm are observed, corresponding to ^1^G_4_ → ^3^F_4_ and ^3^F_3_ → ^3^H_6_ transitions, respectively (in addition, a weak ultraviolet band at ~361 nm is discernible and can be attributed to the ^1^D_2_ → ^3^H_6_ transition) [30,31]. These emissions originate from the strong absorption of 980 nm photons by Yb^3+^ and the subsequent stepwise energy transfer to Tm^3+^, which populates the excited states of Tm^3+^ and enables NIR-to-Vis/UV spectral conversion through multiphoton upconversion processes. The invariant peak positions among different architectures indicate that introducing Au and Al and assembling the multilayer stacks do not perturb the 4f energy-level structure of Tm^3+^. The upconversion emission therefore remains dominated by the intrinsic 4*f*–4*f* transitions of NaYF_4_:Yb^3+^,Tm [36]. Notably, the emission intensity of the 475 nm fluorescence peak increases monotonically with increasing structural complexity, following the order: Al/NaYF_4_:Yb^3+^,Tm^3+^/Au/TiO_2_ > NaYF_4_:Yb^3+^,Tm^3+^/Au/TiO_2_ > NaYF_4_:Yb^3+^,Tm^3+^/TiO_2_. This enhancement can be attributed to the cooperative interaction between the F-P and plasmonic resonances. The F-P cavity enhances both the 980 nm excitation field and the upconverted emission by forming standing-wave modes and effectively extending the optical path length within the NaYF_4_:Yb^3+^,Tm^3+^ layer. Meanwhile, the localized surface plasmon resonance supported by the ultrathin Au layer amplifies the local electromagnetic field, thereby strengthening near-infrared absorption by the NaYF_4_:Yb^3+^,Tm^3+^ particles and improving the radiative out-coupling efficiency of the upconverted photons.

The absorption spectra of different architectures are shown in Figure 3b. As shown in Figure 3b, we investigated the cavity configuration incorporating an Al back reflector and further compared it with a corresponding structure using flexible Al foil as the back mirror. Compared with a relatively smooth vacuum-deposited Al film, the Al foil not only provides higher mirror-like reflectivity but also introduces stronger diffuse reflection and multiple scattering owing to its micro-wrinkled/rough surface. This is expected to further enhance light trapping and extend the effective optical path length within the cavity, thereby improving photon utilization over a broader spectral range. Pristine TiO_2_ films show pronounced absorption mainly in the ultraviolet region, which is consistent with the wide bandgap of TiO_2_. After introducing a Au layer, a distinct extinction enhancement emerges in the visible region due to the excitation of localized surface plasmon resonance, thereby effectively broadening the spectral response [26,37]. Quantitative analysis indicates that the average optical absorptance of the Au/TiO_2_ structure over 200–1000 nm increases from 10.74% for the TiO_2_ film to 29.49%, corresponding to an approximately 1.74-fold enhancement. Upon further incorporating the upconversion layer and the Al back reflector to construct an F-P cavity, the average absorptance of Al/NaYF_4_:Yb^3+^,Tm^3+^/Au/TiO_2_ reaches 42.16%, representing a 2.92-fold increase relative to the TiO_2_ film and a further ~42% improvement compared with the Au/TiO_2_ structure. Among all samples, the flexible Al foil/NaYF_4_:Yb^3+^,Tm^3+^/Au/TiO_2_ exhibits the highest average absorptance (60.57%), which is ~43% higher than that of the rigid Al/NaYF_4_:Yb^3+^,Tm^3+^/Au/TiO_2_ counterpart, and delivers the broadest and strongest absorption window from 300 to 1000 nm. This superiority can be attributed to the micro-wrinkled surface of the flexible Al foil, which introduces additional light scattering and multiple reflections compared with the relatively smooth deposited Al layer, thereby enhancing light trapping and effective absorption [38,39]. Photographs of the rigid Al/NaYF_4_:Yb^3+^,Tm^3+^/Au/TiO_2_ and flexible Al foil/NaYF_4_:Yb^3+^,Tm^3+^/Au/TiO_2_ samples are provided in Figure S7 (Supporting Information). To elucidate the light-field regulation mechanism in the constructed Al/NaYF_4_:Yb^3+^,Tm^3+^/Au/TiO_2_ multilayer, FDTD simulations were performed to analyze the electric-field distributions at different wavelengths. Under 400 nm irradiation, the electric field is mainly distributed within the NaYF_4_:Yb^3+^,Tm^3+^ layer and near the Au/TiO_2_ interface, indicating that the F–P cavity formed by the Al and Au layers can effectively regulate the propagation and localization of visible light within the cavity while generating interfacial field enhancement, which is favorable for subsequent charge separation and interfacial reactions. In contrast, under 980 nm irradiation, the electric field is more distinctly confined within the NaYF_4_:Yb^3+^,Tm^3+^ layer, suggesting that the cavity structure can effectively trap near-infrared light and strengthen its interaction with the upconversion layer, thereby promoting near-infrared absorption and energy transfer in the Yb^3+^/Tm^3+^ system. These results demonstrate that the structural design of the upconversion–plasmonic Fabry–Pérot cavity can simultaneously enhance near-infrared harvesting and interfacial light-field coupling, in good agreement with the original design rationale.

In summary, the as-constructed Al/NaYF_4_:Yb^3+^,Tm^3+^/Au/TiO_2_ cascaded upconversion–plasmonic F–P cavity delivers a synergistic optical gain pathway of “light trapping–spectral conversion–near-field amplification.” The Al back mirror markedly extends the effective optical path length through multiple round-trip reflections and interference, selectively amplifying the cavity modes. The NaYF_4_:Yb^3+^,Tm^3+^ layer upconverts 980 nm near-infrared photons into higher-energy emissions capable of exciting TiO_2_. Meanwhile, the ultrathin Au layer sustains localized optical resonances that enhance extinction in the visible–near-infrared region and generate intense interfacial near-field hotspots. Through the coupling of these three components, the electric field is directionally localized and energy is concentrated at key interfaces across different wavelengths, thereby significantly broadening and strengthening the overall spectral response and providing a solid photon-management basis for the subsequent enhancement of photocatalytic hydrogen evolution.

### 3.4. Photocatalytic Hydrogen-Evolution Performance and Mechanistic Study

**Figure 4:**
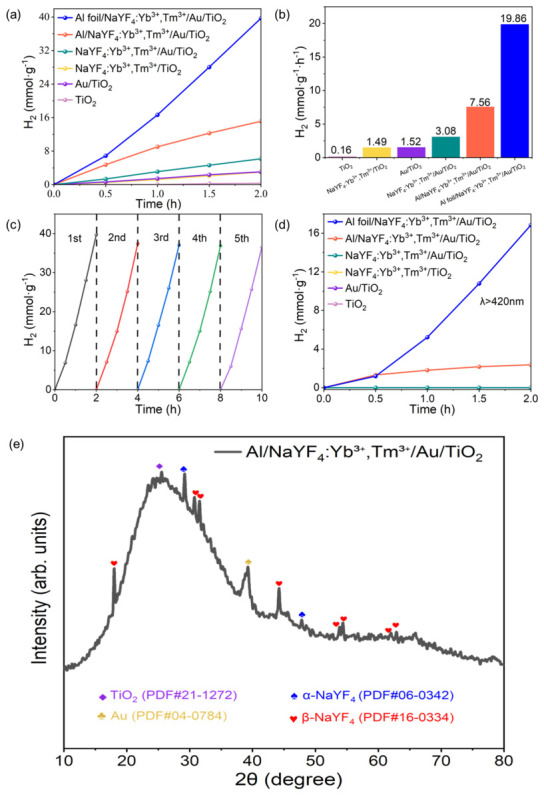
Photocatalytic H_2_−evolution performance and stability of TiO_2_, NaYF_4_:Yb^3+^,Tm^3+^/TiO_2_, Au/TiO_2_, NaYF_4_:Yb^3+^,Tm^3+^/Au/TiO_2_, Al/NaYF_4_:Yb^3+^,Tm^3+^/Au/TiO_2_, and Al foil/NaYF_4_:Yb^3+^,Tm^3+^/Au/TiO_2_. (**a**) Time-dependent cumulative H_2_ yield under identical reaction conditions. (**b**) Corresponding H_2_−evolution rates. (**c**) Cycling stability of the best-performing sample over five consecutive runs. (**d**) Time-dependent cumulative H_2_ yield measured under a 420 nm long−pass filter (λ > 420 nm). (**e**) XRD pattern of the reused Al foil/NaYF_4_:Yb^3+^,Tm^3+^/Au/TiO_2_ photocatalyst after cycling photocatalytic H_2_ evolution.

Figure 4 systematically compares the photocatalytic H_2_-evolution performance of different architectures in an aqueous LA solution under ambient pressure and 300 W Xe lamp irradiation. Figure 4a presents the time-dependent cumulative H_2_ production (all values rounded to two decimals). At 0.5, 1.0, 1.5, and 2.0 h, the cumulative H_2_ yields of TiO_2_ are 0.00, 0.00, 0.23, and 0.32 mmol·g^−1^, respectively. The corresponding values for NaYF_4_:Yb^3+^,Tm^3+^/TiO_2_ are 0.51, 1.21, 2.10, and 2.99 mmol·g^−1^, for Au/TiO_2_ are 0.65, 1.44, 2.36, and 3.04 mmol·g^−1^, for NaYF_4_:Yb^3+^,Tm^3+^/Au/TiO_2_ are 1.34, 3.10, 4.63, and 6.17 mmol·g^−1^, for Al/NaYF_4_:Yb^3+^,Tm^3+^/Au/TiO_2_ are 4.76, 9.02, 12.27, and 15.12 mmol·g^−1^, and for Al foil/NaYF_4_:Yb^3+^,Tm^3+^/Au/TiO_2_ are 6.88, 16.65, 28.05, and 39.72 mmol·g^−1^, respectively. A clear increase in the slope of the curves is observed as the architecture evolves from TiO_2_ to structures incorporating the upconversion layer, the Au layer, and the Al back reflector, indicating a steady acceleration of H_2_ evolution. Notably, the flexible Al-foil-based system delivers a cumulative H_2_ yield of 39.72 mmol·g^−1^ at 2 h, which is substantially higher than that of the rigid Al-based counterpart (15.12 mmol·g^−1^), suggesting that the micro-wrinkled Al foil provides additional scattering/multiple reflections that further strengthen light trapping and increase the effective reaction photon flux. Furthermore, Figure 4b summarizes the H_2_ evolution rates: 0.16 mmol·g^−1^·h^−1^ for TiO_2_, 1.49 mmol·g^−1^·h^−1^ for NaYF_4_:Yb^3+^,Tm^3+^/TiO_2_ (about 9 times relative to TiO_2_), 1.52 mmol·g^−1^·h^−1^ for Au/TiO_2_ (about 9 times relative to TiO_2_), 3.08 mmol·g^−1^·h^−1^ for NaYF_4_:Yb^3+^,Tm^3+^/Au/TiO_2_ (about 19 times relative to TiO_2_), 7.56 mmol·g^−1^·h^−1^ for Al/NaYF_4_:Yb^3+^,Tm^3+^/Au/TiO_2_ (about 47 times relative to TiO_2_), and 19.86 mmol·g^−1^·h^−1^ for Al foil/NaYF_4_:Yb^3+^,Tm^3+^/Au/TiO_2_ (about 124 times relative to TiO_2_). Together, the cumulative-yield and rate data corroborate each other, demonstrating that upconversion-assisted spectral conversion, Au-plasmon-enhanced light harvesting, and Al back-reflection/cavity effects act cooperatively in a cascaded manner to improve Vis–NIR photon utilization and translate it into accelerated H_2_-evolution kinetics.

By correlating the optical results in Figure 3 with the H_2_-evolution performance in Figure 4, it is evident that the stepwise enhancement in hydrogen evolution is highly consistent with the cascaded photon-management pathway of “enhanced absorption–enhanced upconversion–enhanced near-field.” Pristine TiO_2_ absorbs predominantly in the ultraviolet region, resulting in limited photon utilization and thus the lowest H_2_-evolution rate and cumulative H_2_ yield. After introducing NaYF_4_:Yb^3+^,Tm^3+^, the upconversion emission under 980 nm excitation generates blue/near-UV photons, providing an additional excitation channel for TiO_2_ beyond its intrinsic absorption range and thereby markedly improving H_2_ production. Upon further incorporation of an ultrathin Au layer, visible-region extinction is significantly enhanced due to localized surface plasmon resonance, while the intensified electromagnetic near field promotes upconversion excitation and interfacial energy coupling, leading to a further increase in H_2_ output. Finally, when an Al back reflector—particularly the micro-wrinkled flexible Al foil—is introduced to form an F–P cavity, multiple round-trip reflections/interference substantially extend the effective optical path length and strengthen the intracavity field. This simultaneously boosts the upconversion emission intensity and broadband absorption, enabling more visible–near-infrared photons to be converted and efficiently coupled to the TiO_2_ reaction interface, thus delivering the highest H_2_-evolution kinetics and the largest 2 h cumulative H_2_ yield. Overall, the broadband absorption enhancement and the stepwise amplification of upconversion emission provide direct optical evidence for the pronounced improvement in hydrogen-evolution performance. To further place our results in the context of existing photocatalytic H_2_-evolution systems, the H_2_-evolution performance of the optimized Al foil/NaYF_4_:Yb^3+^,Tm^3+^/Au/TiO_2_ catalyst was compared with recently reported representative photocatalysts under comparable 300 W Xe-lamp irradiation conditions, as summarized in Table S1 (Supporting Information) [40,41,42,43,44,45,46,47,48,49,50]. The optimized catalyst exhibits a competitive H_2_-evolution rate of 19.86 mmol·g^−1^·h^−1^, highlighting the effectiveness of the cascaded photon-management strategy involving upconversion-assisted spectral conversion, Au-plasmon-enhanced light harvesting, and Al-assisted Fabry–Pérot cavity light trapping.

The cycling stability of the optimized architecture was further evaluated, as shown in Figure 4c. Under five consecutive on–off illumination cycles, the H_2_ evolution rate of Al foil/NaYF_4_:Yb^3+^,Tm^3+^/Au/TiO_2_ remains essentially unchanged, demonstrating excellent cycling stability and structural durability. This result indicates that the triple optical coupling inherent to the Al/NaYF_4_:Yb^3+^,Tm^3+^/Au/TiO_2_ design is reproducible under operating conditions and does not introduce appreciable deactivation pathways.

To further verify the structural stability of the photocatalyst, the reused Al foil/NaYF_4_:Yb^3+^,Tm^3+^/Au/TiO_2_ sample after the cycling photocatalytic H_2_ evolution test was collected and characterized by XRD. As shown in Figure 4e, the reused photocatalyst still exhibits the characteristic diffraction peaks corresponding to TiO_2_, Au, α-NaYF_4_, and β-NaYF_4_. Compared with the XRD pattern of the fresh sample shown in Figure 2a, no obvious impurity peaks or phase transformation can be observed after repeated reaction. This result indicates that the main crystalline phases of the multilayer photocatalyst are well maintained, confirming its good structural stability during PLA wastewater reforming for H_2_ production.

To assess the contribution of hot electrons, long-pass filter experiments (λ > 420 nm) were carried out, as shown in Figure 4d. Under this condition, TiO_2_ is intrinsically limited by its bandgap (e.g., 3.2 eV), with its absorption mainly concentrated in the ultraviolet region below 390 nm; therefore, after removing the UV component, its band-to-band excitation is significantly suppressed. Notably, the samples with the upconversion-plasmonic F-P cavity architecture still retain appreciable H_2_-evolution activity under filtered illumination. Specifically, the cumulative H_2_ yields of Al/NaYF_4_:Yb^3+^,Tm^3+^/Au/TiO_2_ at 0.5, 1.0, 1.5, and 2.0 h are 1.32, 1.81, 2.17, and 2.37 mmol·g^−1^, respectively, whereas those of Al foil/NaYF_4_:Yb^3+^,Tm^3+^/Au/TiO_2_ reach 1.19, 5.22, 10.79, and 16.85 mmol·g^−1^, respectively. In contrast, the control samples (e.g., TiO_2_, Au/TiO_2_, and NaYF_4_:Yb^3+^,Tm^3+^/TiO_2_) show no detectable H_2_ evolution under the same filtered condition, indicating that plasmon-induced hot-electron injection driven solely by visible-light excitation is insufficient to provide effective charge carriers of adequate quantity/energy without efficient coupling and field enhancement. These results demonstrate that the proposed cascade photon upcycling strategy can intensify NIR absorption and upconversion within the NaYF_4_:Yb^3+^,Tm^3+^ layer via multiple round-trip reflections in the F-P cavity, converting low-energy NIR photons into higher-energy UV/Vis emissions capable of exciting TiO_2_. Together with the plasmonic near-field enhancement of the Au layer, this synergy improves energy coupling and carrier injection efficiency, thereby enabling stable and pronounced H_2_ evolution even under non-UV (Vis-NIR) illumination.

**Figure 5:**
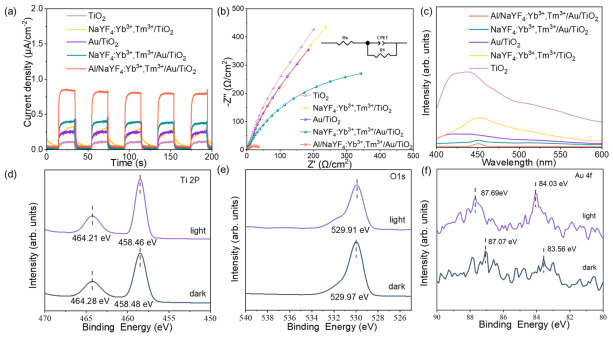
Photoelectrochemical characterization of the Al/NaYF_4_:Yb^3+^,Tm^3+^/Au/TiO_2_ multilayer. (**a**) Transient photocurrent responses under on/off light switching. (**b**) EIS Nyquist plots under illumination with the corresponding equivalent electrical circuit shown in the inset. (**c**) Steady−state PL spectra of the TiO_2_ layers for five architectures (λ_ex_ = 365 nm). (**d**–**f**) In situ XPS collected after 10 min in the dark and after 10 min under full−spectrum (200−2500 nm) illumination: (**d**) Ti 2*p*, (e) O 1*s*, and (**f**) Au 4*f*.

As shown in Figure 5a, transient photocurrent responses of the samples were recorded in a 0.5 M Na_2_SO_4_ electrolyte under 300 W Xe-lamp irradiation. Among all the samples, Al/NaYF_4_:Yb^3+^,Tm^3+^/Au/TiO_2_ exhibits the highest photocurrent density together with sharp and reproducible on/off switching behavior, indicating more efficient photogenerated charge-carrier generation and transport. In contrast, pristine TiO_2_ and NaYF_4_:Yb^3+^,Tm^3+^/TiO_2_ display much weaker photocurrent responses, reflecting their limited light-utilization capability and inefficient carrier extraction. To further probe the interfacial charge-transfer characteristics under illumination, electrochemical impedance spectroscopy (EIS) was performed. As shown in Figure 5b, the corresponding equivalent electrical circuit is shown in the inset and can be expressed as R_s_−(R_1_//CPE_1_), where R_s_ represents the solution resistance, R_1_ corresponds to the interfacial charge-transfer resistance, and CPE_1_ represents the constant phase element associated with the non-ideal capacitive behavior at the electrode/electrolyte interface. The Nyquist plots reveal that Al/NaYF_4_:Yb^3+^,Tm^3+^/Au/TiO_2_ possesses the smallest semicircle diameter among all the samples, indicating the lowest interfacial charge-transfer resistance. This result suggests faster interfacial charge transport and weaker carrier recombination, both of which are essential for sustaining high photocatalytic activity. The reduced impedance can be ascribed to the enhanced carrier mobility and improved utilization of photogenerated charges enabled by the coupled NaYF_4_:Yb^3+^,Tm^3+^-plasmon-cavity architecture. To further evaluate the charge-recombination behavior, steady-state photoluminescence (PL) measurements were carried out for all the samples, as shown in Figure 5c. Pristine TiO_2_ exhibits strong PL emission, indicative of severe radiative recombination. With the introduction of NaYF_4_:Yb^3+^,Tm^3+^ particles and the Au interlayer, the PL intensity gradually decreases. Notably, Al/NaYF_4_:Yb^3+^,Tm^3+^/Au/TiO_2_ shows the lowest PL intensity, demonstrating the most effective suppression of radiative recombination. This pronounced PL quenching originates from enhanced light absorption, plasmon-assisted energy redistribution, and cavity-induced modulation of the local optical field, which collectively promote charge separation and interfacial charge utilization rather than radiative decay. Combined with the significantly enhanced H_2_-evolution rate and cumulative H_2_ yield shown in Figure 4, these results demonstrate that the cascaded coupling in the Al/NaYF_4_:Yb^3+^,Tm^3+^/Au/TiO_2_ cavity not only broadens the spectral response and strengthens the localized optical field, but also simultaneously promotes charge-carrier generation, separation, and interfacial migration. As a result, the improved photon utilization is effectively translated into superior photocatalytic hydrogen-evolution performance. As shown in Figure 5d–f, the in situ XPS spectra recorded under dark and light-irradiation conditions reveal differentiated shifts in the core-level binding energies of the constituent elements. It should be noted that these in situ XPS measurements were performed to monitor the light-induced binding-energy shifts rather than to quantify the relative proportions of different chemical states; therefore, the spectra are discussed based on the peak-position variations without further peak deconvolution. Specifically, the Ti 2*p* peaks shift overall toward lower binding energy after light irradiation, with Ti 2*p*_3/2_ moving from 458.48 to 458.46 eV and Ti 2*p*_1/2_ from 464.28 to 464.21 eV, corresponding to negative shifts of approximately 0.02 eV and 0.07 eV, respectively. Meanwhile, the O 1*s* peak shifts from 529.97 to 529.91 eV, showing a negative shift of about 0.06 eV. These negative shifts indicate an increased electron density on the TiO_2_ side, i.e., photoinduced electron accumulation. In contrast, the Au 4*f* peaks shift toward higher binding energy under illumination, with Au 4*f*_7/2_ moving from 83.56 to 84.03 eV and Au 4*f*_5/2_ from 87.07 to 87.69 eV, corresponding to positive shifts of approximately 0.47 eV and 0.62 eV, respectively. The positive shift of Au 4*f* indicates a decrease in electron density in the Au layer, suggesting that Au is in an electron-deficient state. Therefore, the opposite binding-energy shifts of Ti/O and Au demonstrate that electrons migrate from the Au layer to the TiO_2_ side during illumination, with Au serving as the electron-donating/output side and TiO_2_ as the electron-accepting/accumulation side. Combined with the optical design of the multilayer structure and the H_2_-evolution results, it can be further inferred that the Au/TiO_2_ interface acts as the key charge-separation and charge-transfer interface. Under the combined effects of localized surface plasmon excitation and cavity-field enhancement, the Au layer generates and delivers energetic electrons, which are injected into and accumulated on the TiO_2_ surface, thereby increasing the electron supply required for surface reduction reactions. Accordingly, the Ti-related surface sites of TiO_2_ and their neighboring Ti-O structural units are considered to be the main H_2_-evolution reaction sites, whereas Au primarily functions in light harvesting, energetic-electron generation, and interfacial electron transport. Overall, the in situ XPS results reveal a clear Au → TiO_2_ directional electron-transfer pathway in this system, indicating that the Au/TiO_2_ interface is the core active region where photogenerated charge separation is coupled with surface catalysis, while the TiO_2_ surface serves as the principal site for proton reduction and hydrogen evolution [51].

## 4. Conclusions

In summary, an upconversion–plasmonic Fabry–Pérot cavity (Al/NaYF_4_:Yb^3+^,Tm^3+^/Au/TiO_2_) featuring cascaded light-energy regulation was successfully constructed for efficient broadband solar-energy utilization in photocatalytic hydrogen production from PLA wastewater. By rationally integrating upconversion nanoparticles, an ultrathin plasmonic Au layer, and an Al back reflector into a single multilayer architecture, the system establishes a cascaded light-utilization pathway involving near-infrared capture and upconversion, visible-light plasmonic enhancement, and cavity-resonance coupling. The NaYF_4_:Yb^3+^,Tm^3+^ layer converts low-energy near-infrared photons into higher-energy emissions, thereby extending the photoresponse of TiO_2_ beyond its intrinsic ultraviolet region. Meanwhile, the plasmonic Au layer enhances visible-light extinction and facilitates interfacial energy and charge transfer, while the Al back reflector, together with the upper metal layer, forms a Fabry–Pérot cavity that further improves optical-field confinement and photon utilization by extending the optical path length and regulating resonant modes. As a result, the system enables coordinated utilization of near-infrared, visible, and ultraviolet light, which is ultimately translated into enhanced interfacial charge separation and improved reaction kinetics. Accordingly, the Al/NaYF_4_:Yb^3+^,Tm^3+^/Au/TiO_2_ architecture achieves a hydrogen-evolution rate of 7.56 mmol·g^−1^·h^−1^ from PLA wastewater under simulated solar irradiation, which is about 47 times that of pristine TiO_2_. After the introduction of flexible Al foil, the Al foil/NaYF_4_:Yb^3+^,Tm^3+^/Au/TiO_2_ structure delivers a hydrogen-evolution rate of 19.86 mmol·g^−1^·h^−1^, approximately 124 times that of pristine TiO_2_, together with good cycling stability. Photoelectrochemical measurements, photoluminescence analysis, and in situ XPS collectively indicate that the enhanced performance does not arise from ultraviolet excitation alone, but rather from the cooperative harvesting of broadband light, suppressed charge-carrier recombination, and promoted interfacial charge transport enabled by the cascaded structure.

This work demonstrates that the rational integration of upconversion, plasmonic enhancement, and cavity resonance within a single multilayer system offers an effective structural strategy for cascaded utilization of broadband solar energy. Beyond PLA-derived wastewater, this strategy may be extended to selected plastic-derived substrates, particularly polymers containing hydrolysable or oxygen-containing groups. However, it should not be regarded as directly applicable to all plastics, especially chemically inert and highly hydrophobic polymers such as polyethylene, polypropylene, and polystyrene, which may require additional pretreatment before photocatalytic conversion. Further studies are therefore needed to evaluate its applicability to different plastic waste streams.

## Figures and Tables

**Table 1:** Crystallite sizes of the individual crystalline components calculated from XRD data.

Phase	Selected Peak	2θ/°	FWHM/°	Crystallite Size/nm
TiO_2_	TiO_2_ (101)	25.00	1.235	6.6
Au	Au (111)	38.90	1.15	7.3
α-NaYF_4_:Yb^3+^,Tm^3+^	representative peak	28.31	0.147	55.6
β-NaYF_4_:Yb^3+^,Tm^3+^	representative peak	30.70	0.155	53.2

## Data Availability

The original contributions presented in this study are included in the article/Supplementary Material. Further inquiries can be directed to the corresponding authors.

## Supplementary Materials

The following supporting information can be downloaded at: https://www.mdpi.com/article/10.3390/ma19142994/s1.
