# Supplementary material for: Cascaded Photon Upcycling in an Upconversion-Plasmonic Fabry-Pérot Cavity for Broadband Solar Hydrogen Production from PLA Waste
Source: Materials (Basel). 2026 Jul 11;19(14):2994. doi: 10.3390/ma19142994 (PMC13413013; doi:10.3390/ma19142994)
Supplement: Supplementary file 1 [file materials-19-02994-s001.zip › materials-4398136-supplementary.pdf]

Supporting Information

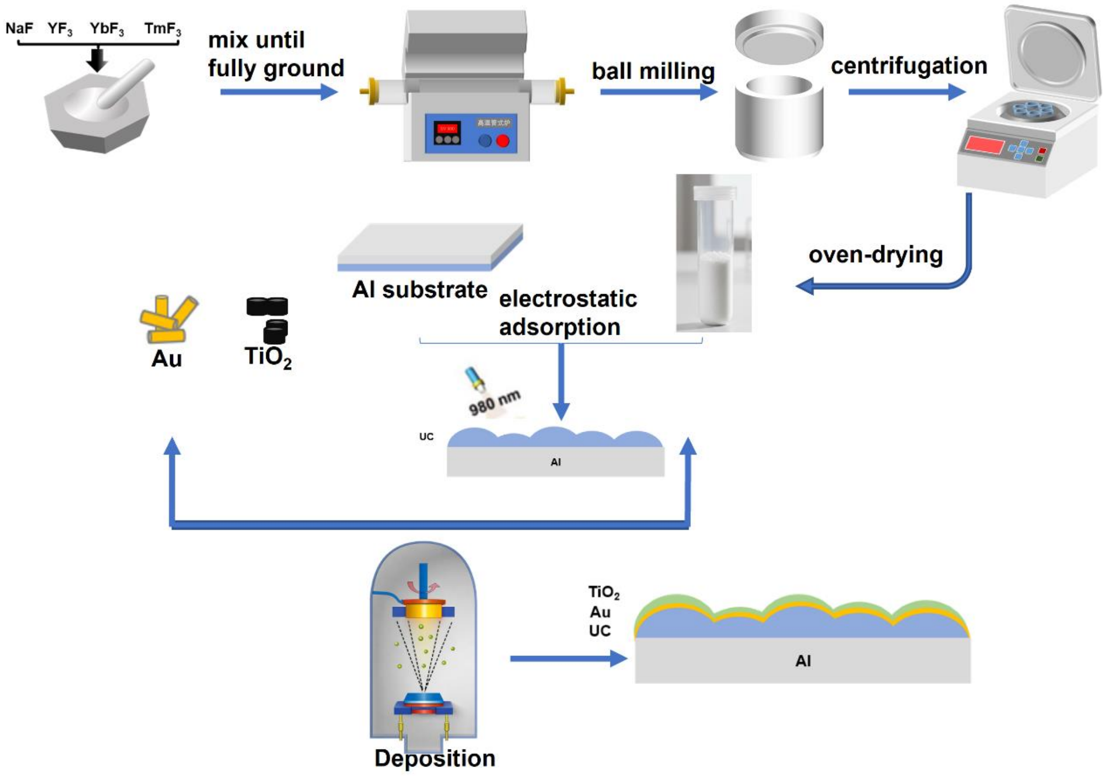

Figure S1. Schematic workflow for preparing the $NaYF_4:Yb^{3+},Tm^{3+}$ upconversion powder and the $Al/NaYF_4:Yb^{3+},Tm^{3+}/Au/TiO_2$ multilayer structure.

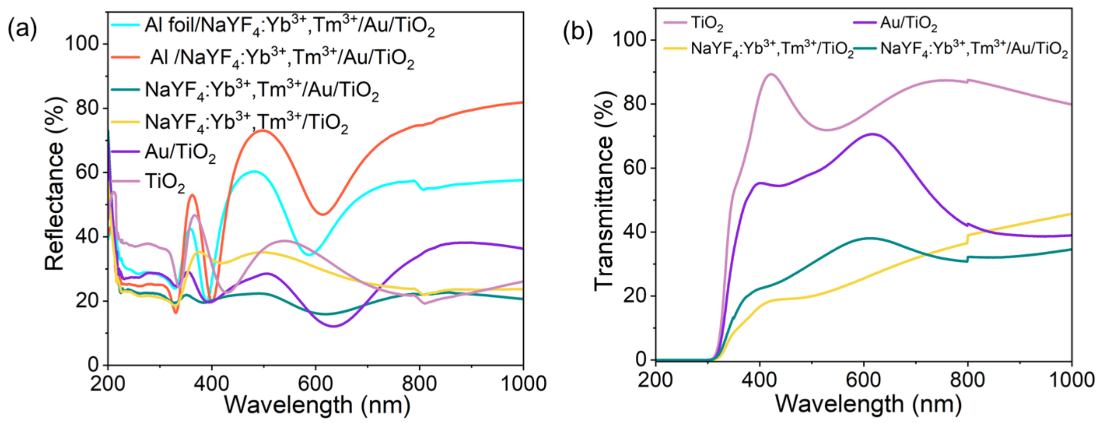

Figure S2. (a) Reflectance spectra (200–1000 nm) of different samples: TiO_2, $NaYF_4$:Yb^{3+},Tm^{3+}/TiO_2, Au/TiO_2, $NaYF_4$:Yb^{3+},Tm^{3+}/Au/TiO_2, Al/$NaYF_4$:Yb^{3+},Tm^{3+}/Au/TiO_2, and Al foil/ $NaYF_4$:Yb^{3+},Tm^{3+}/Au/TiO_2. (b) Transmittance spectra (200–1000 nm) of different samples: TiO_2, $NaYF_4$:Yb^{3+},Tm^{3+}/TiO_2, Au/TiO_2, and $NaYF_4$:Yb^{3+},Tm^{3+}/Au/TiO_2.

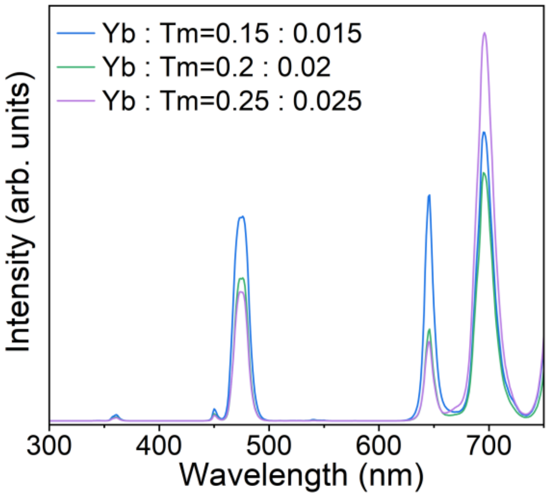

Figure S3. Upconversion emission spectra of $NaYF_4$:Yb^{3+},Tm^{3+} phosphors with different doping ratios under 980 nm excitation(λ_{ex} = 980nm).

With increasing milling time from 0 to 6 h, the intensities of the main diffraction peaks gradually decrease, accompanied by an evident broadening of the full width at half maximum (FWHM). This evolution reflects the combined effects of crystallite-size refinement and the accumulation of lattice microstrain induced by mechanical impact. For completeness, the XRD patterns of all architectures as well as $NaYF_4$:Yb^{3+},Tm^{3+} powders with different milling durations are provided in Fig. S2 (Supporting Information).

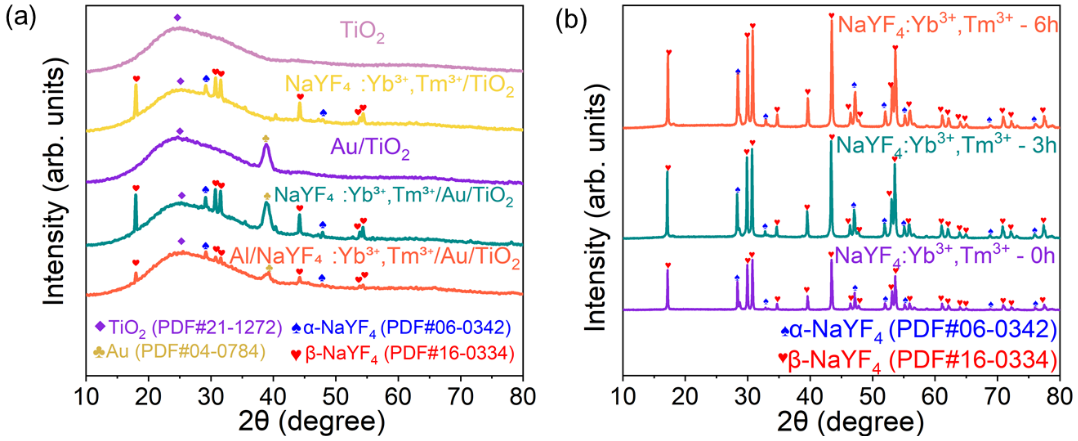

Figure S4. (a) XRD patterns of TiO_2, $NaYF_4$:Yb^{3+},Tm^{3+}/TiO_2, Au/ TiO_2, $NaYF_4$:Yb^{3+},Tm^{3+}/Au/ TiO_2, and Al/$NaYF_4$:Yb^{3+},Tm^{3+}/Au/TiO_2.(b) XRD patterns of $NaYF_4$:Yb^{3+},Tm^{3+} powders with different ball-milling durations.

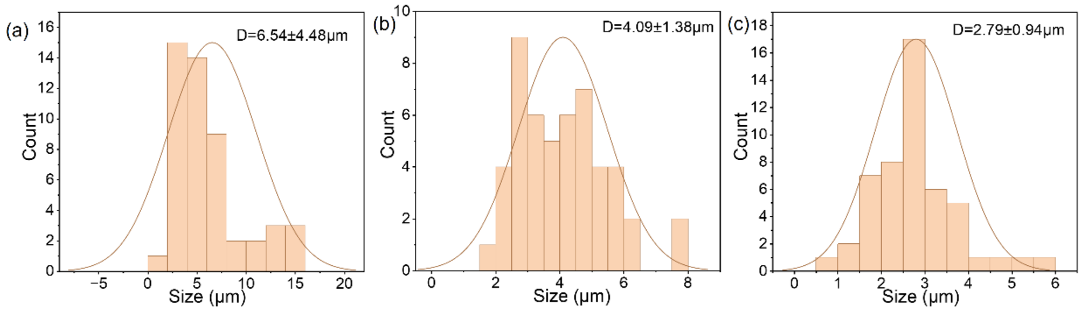

Figure S5. Particle size distribution histograms of $NaYF_4$:Yb^{3+},Tm^{3+} powders at different ball-milling durations: (a) $NaYF_4$:Yb^{3+},Tm^{3+} - 0 h, (b) $NaYF_4$:Yb^{3+},Tm^{3+} - 3 h, and (c) $NaYF_4$:Yb^{3+},Tm^{3+} - 6 h.

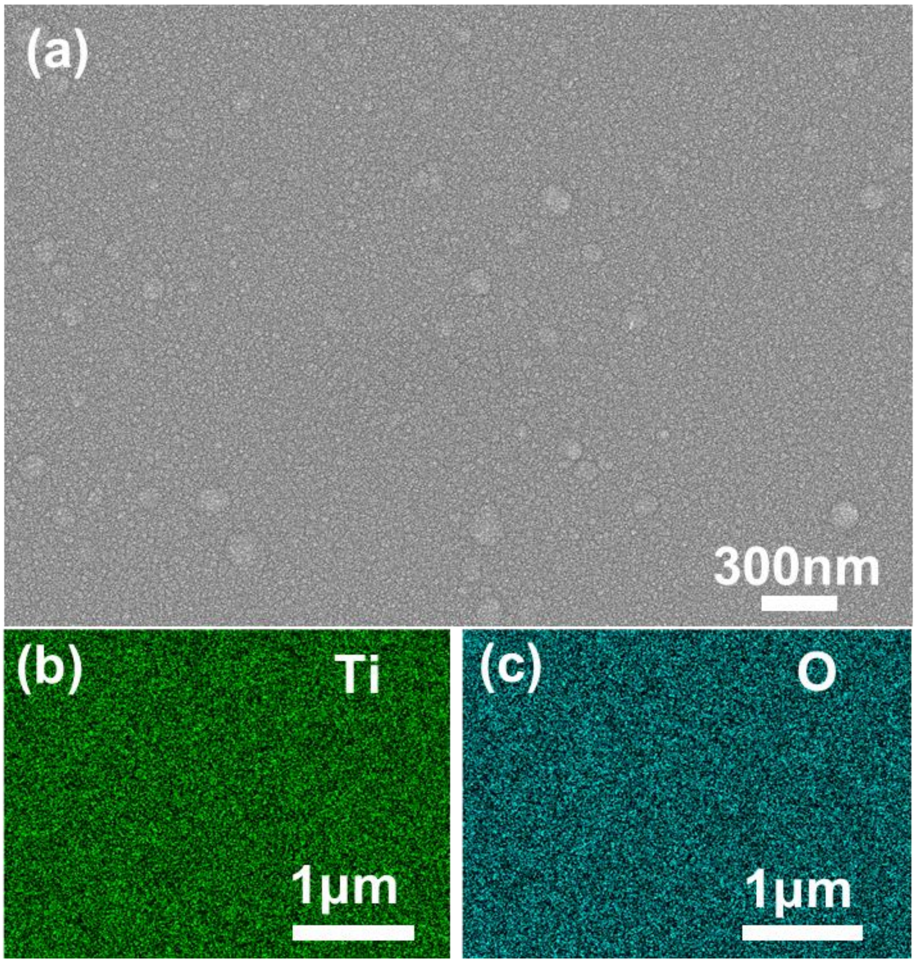

Figure S6. SEM image and corresponding EDS elemental mapping of the sample: (a) SEM image; (b) Ti elemental map; (c) O elemental map.

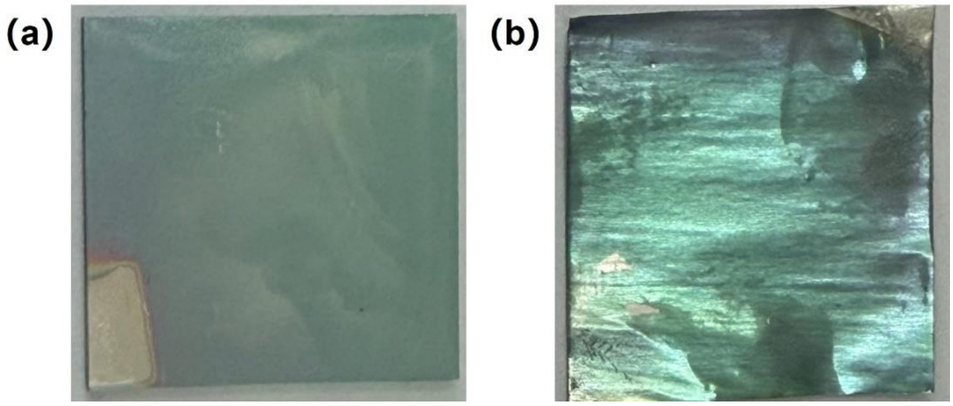

Figure S7. Photographs of the two multilayer-structured samples: (a) Al/$NaYF_4$:Yb^{3+},Tm^{3+}/Au/TiO_2 structure and (b) Al foil/$NaYF_4$:Yb^{3+},Tm^{3+}/Au/TiO_2 structure.

Table S1. Photocatalytic H_2 evolution performance of various photocatalysts.

Samples	Light source	Hydrogen evolution (mmol h-1 g-1)	Reference
Al foil/ $NaYF_4$:Yb^{3+},Tm^{3+}/Au/ TiO_2	300W Xe Lamp	19.85	This work
Ni/TiO_2	300W Xe Lamp	2.416	[1]
Ag-Cu/TiO_2	300W Xe Lamp	0.26	[2]
Au NR-TiO_2	300W Xe Lamp	2.21	[3]
h-ZnSe/Pt@TiO_2	300W Xe Lamp	1.86	[4]
CdS/Au-Ag/B-TiO_2	300W Xe Lamp	15.97	[5]
Ni/TiO_2	300W Xe Lamp	1.60	[6]
Ag mono@TiO_2-Au	300W Xe Lamp	5.00	[7]
$Pt_{0.5}$-$Au_{1.5}$/CN	300W Xe lamp	3.18	[8]
Pt1-HCN (0.38%) -125	300W Xe Lamp	2.90	[9]
CdS/(Pt/g-C_3N_4)	300W Xe Lamp	1.46	[10]
Au/TiO_2-g-C_3N_4- (95-5)	300W Xe Lamp	18.39	[11]

References:

[1] Wang, Z., Huang, X., Jia, Y., Guo, L., Wang, H., & Dai, W. Localized surface plasmon resonance-induced bidirectional electron transfer of formic acid adsorption for boosting photocatalytic hydrogen production on Ni/TiO_2. *Chem. Eng. J. 2024, 482*, 148942.
[2] Mani, S. S., Rajendran, S., Arun, P. S., Vijaykumar, A., Mathew, T., & Gopinath, C. S. Bimetallic and plasmonic Ag and Cu integrated TiO_2 thin films for enhanced solar hydrogen production in direct sunlight. *Energy Adv. 2024, 3*(4), 829-840.
[3] Ye, Z., Lu, P., Chen, Y., Xu, Z., Huang, H., Zhi, M., ... & Yan, B. Synthesis and photocatalytic property of Au–TiO_2 nanocomposites with controlled morphologies in microfluidic chips. *Lab Chip 2024, 24*(8), 2253-2261.

[4] You, Y., Chen, S., Zhao, J., Lin, J., Wen, D., Sha, P., ... & Huang, S. Rational design of S-scheme heterojunction toward efficient photocatalytic cellulose reforming for H_2 and formic acid in pure water. *Adv. Mater. 2024, 36*(4), 2307962.
[5] Li, Q., Yang, S., Liu, R., Huang, Y., Liang, Y., Hu, C., ... & Li, Y. Synergetic effect of the interface electric field and the plasmon electromagnetic field in Au-Ag alloy mediated Z-type heterostructure for photocatalytic hydrogen production and CO_2 reduction. *Appl. Catal. B Environ. 2023, 331*, 122700.
[6] Birla, P. N., Arbuj, S., Shinde, M. D., Joseph, S., Rane, S., Kulkarni, S., & Kale, B. Electroless Ni plated nanostructured TiO_2 as a photocatalyst for solar hydrogen production. *RSC Adv. 2023, 13*(29), 20068-20080.
[7] Liu, M., Jin, X., Li, S., Billeau, J. B., Peng, T., Li, H., ... & Zhang, J. Enhancement of scattering and near field of TiO_2–Au nanohybrids using a silver resonator for efficient plasmonic photocatalysis. *ACS Appl. Mater. Interfaces 2021, 13*(29), 34714-34723.
[8] Ding, F., Yu, H., Liu, W., Zeng, X., Li, S., Chen, L., ... & Wu, C. Au-Pt heterostructure cocatalysts on g-C_3N_4 for enhanced H2 evolution from photocatalytic glucose reforming. *Mater. Des. 2024, 238*, 112678.
[9] Mahvelati-Shamsabadi, T., Bhamu, K. C., Lee, S. H., Dang, T. T., Khoi, V. H., Hur, S. H., ... & Chung, J. S. Coordinatively unsaturated atomically dispersed Pt^{+}_2-N_4 sites on hexagonal nanosheet structure of g-C_3N_4 for high-performance photocatalytic H2 production. *Appl. Catal. B Environ. 2023, 337*, 122959.
[10] Hu, W., Zhang, J., Tian, J., Dang, Y., Wang, W., Zhan, H., & Ma, B. Greatly enhanced photocatalytic hydrogen production on CdS/(Pt/g-C_3N_4) via dual functions of Pt on semiconductors interface. *Int. J. Hydrogen Energy 2022, 47*(68), 29295-29304.
[11] Jiménez-Calvo, P., Caps, V., Ghazzal, M. N., Colbeau-Justin, C., & Keller, V. Au/TiO_2 (P25)-gC_3N_4 composites with low gC_3N_4 content enhance TiO_2 sensitization for remarkable H_2 production from water under visible-light irradiation. *Nano Energy 2022, 75*, 104888.
